# Feline Coronaviruses Identified in Feline Effusions in Suspected Cases of Feline Infectious Peritonitis

**DOI:** 10.3390/microorganisms9091801

**Published:** 2021-08-24

**Authors:** Shih-Jung Yen, Hui-Wen Chen

**Affiliations:** Department of Veterinary Medicine, National Taiwan University, 1 Sec 4 Roosevelt Road, Taipei 10617, Taiwan; whitney11940@gmail.com

**Keywords:** feline coronavirus, feline infectious peritonitis, immunofluorescence staining, genotype, phylogenetic analysis

## Abstract

Ninety-five effusion samples were collected from cats with suspected feline infectious peritonitis in northern Taiwan; these samples showed a 47.4% (45/95) feline coronavirus (FCoV) positivity rate on immunofluorescence staining and RT-PCR. Young cats (≤24 months old) were found to have a significantly higher risk than cats >24 months old (odds ratio (OR) = 6.19, 95% confidence interval (CI) 2.54–16.00). No significant association was found between the positive rates and sex or breed. The A/G ratio in positive cases was significantly lower than the A/G ratio in negative cases. Genotyping and sequencing of the positive cases revealed 71.9% single infection with type I strains and 28.1% coinfection with types I and II. No single infections with type II strains were noted. The type I sequences had high diversity, while the type II sequences had high internal sequence identity and were more similar to CoVs from other species, such as dogs, pigs, and various small mammals. This study demonstrates the latest analysis of FCoV infection cases in northern Taiwan.

## 1. Introduction

Feline coronavirus (FCoV) is a member of the genus Alphacoronavirus, a group of enveloped, single-stranded, positive-sense RNA viruses [1]. FCoV has two distinct pathotypes named feline enteric coronavirus (FECV) and feline infectious peritonitis virus (FIPV), which cause different pathological symptoms [2]. FECV mainly infects cats through the fecal–oral route and causes mild and transient gastroenteritis and, at times, results in asymptomatic infections. In contrast, FIPV emerges from a mutation of FECV within a small percentage of infected cats, and few horizontal transmissions are observed [3,4]. The mutation and pathogenesis leading to the development of feline infectious peritonitis (FIP) are still unknown [5,6,7]. FIP remains a frustrating systemic disease for veterinarians and pet owners due to the high mortality rate and limited diagnostic and treatment methods [8]. Although it is generally considered that all cats regardless of sex and breed are susceptible to FIP, some reports show that male cats, purebred cats, young cats, and those living in multi-cat households may be predisposed to the disease [4]. The gold standard for a definitive diagnosis of FIP remains immunohistochemistry staining of FCoV within characteristic histopathological tissue lesions. Immunofluorescence staining in cats with effusion is also a promising method of diagnosis, achieving 100% specificity with 57–95% sensitivity in four studies [9,10,11,12], and 71% specificity with 100% sensitivity in another study [13].

Several studies have concluded that FCoV can be subdivided into type I and type II based on serological and genetic differences. The ancestor of type I FCoV is unknown, but it is currently suspected to originate from bats. Type II FCoV originated from the recombination of type I FCoV and canine coronavirus (CCoV) [14]. Although FCoV has a high frequency of mutation, the development of new viral variants seems to be limited in comparison to other species. Some authors deduced that pet cats had relatively infrequent interaction with other animal species, reducing the probability of interaction between FCoV and CoVs from other species; in contrast, wild cats may face more exposure to CoVs due to infection of their prey, specifically birds and rodents.

Two previous epidemiological studies of FCoV in Taiwan have been reported. From 2003 to 2007, Lin et al. reported genotype results indicating 88.7% single infection with type I, 5.9% single infection with type II, and 5.4% coinfection out of 222 cases [14]. Subsequently, we observed a trend of increasing type II strain (19%) and coinfection cases (19%) in the period between September 2017 and January 2019 [15]. The aim of the present study was to monitor FCoV phylogeny in strongly suspected cases of FIP in northern Taiwan and to investigate the genetic diversity of FCoV.

## 2. Materials and Methods

### 2.1. Feline Effusion Samples

Effusion samples from 95 FIP-suspected cats during the period of January 2019 to October 2020 were included in this study. These samples came from local animal hospitals in northern Taiwan and from the National Taiwan University Veterinary Hospital. The effusion types were composed of 60 ascites, 31 pleural effusions, 2 with both ascites and pleural effusion, 1 pericardial effusion, and 1 renal cyst fluid. All the samples underwent indirect immunofluorescence assay (IFA) and reverse transcription polymerase chain reaction (RT-PCR) to detect the FCoV nucleocapsid (N) protein in macrophages and FCoV 3′ untranslated region (UTR) fragments as described below. Sex, breed, age, and serum albumin/globulin ratio (A/G ratio) from these cats were recorded; the age data were missing for 2 cats, and the A/G ratios were missing for 3 cats.

### 2.2. Indirect Immunofluorescence Assay (IFA)

The IFA procedure in this study was based on previously published protocols, with modifications [13,15]. The effusion samples were processed and stored at 4 °C on the day they arrived. First, the samples were washed with PBS, and the cell count was adjusted to 200,000–500,000 cells per 100 µL. The processed samples were then centrifuged at 1000 rpm for 10 min at 4 °C with a cytocentrifuge. The slides were then fixed with 80% acetone at −20 °C. For blocking, the slides were incubated with 10% normal goat serum (Jackson ImmunoResearch, West Grove, PA, USA) at room temperature in a moist box. The mouse anti-FCoV N protein monoclonal antibody (Bio-Rad, MCA2194) was used at a 1:400 dilution in 10% goat serum for 45 min at room temperature as the primary antibody. The slides were washed three times with PBST (PBS containing 0.01% Tween 20), and goat anti-mouse IgG FITC conjugate (Jackson ImmunoResearch) was then added at a 1:400 dilution in 10% goat serum for 30 min at room temperature in the dark as the secondary antibody. Finally, the slides were washed for 15 min and sealed with mounting solution containing DAPI (Vectashield, Burlingame, CA, USA). The slides were then interpreted with a fluorescence microscope (Olympus IX83).

### 2.3. Viral RNA Extraction and RT-PCR

Viral RNA was extracted from each of the effusion samples using the PetNAD Nucleic Acid Co-Prep kit (GeneReach, Taichung, Taiwan) and then reverse-transcribed into complementary DNA (cDNA) using the M-MLV reverse transcriptase protocol (Invitrogen, Carlsbad, CA, USA). We used Herrewegh’s nested PCR method, targeting the 3′ UTR to determine the presence of FCoV RNA, and Addie’s nested PCR method, targeting the partial spike gene for genotyping with modifications [16,17]. GoTaq (Promega, Madison, WI, USA) was used as the PCR reagents following the manufacturer’s instructions. Cycling conditions were identical to those used in the references. For 3′ UTR detection, we used oligo(dT) as the primer and followed the MMLV protocol to reverse transcribe viral RNA to cDNA. The forward primer P205 (5′-GGCAACCCGATGTTTAAAACTGG-3′) and reverse primer P211 (5′-CACTAGATCCAGACGTTAGCTC-3′) were used in the first PCR procedures and formed a 233-bp product. The specific 177-bp final PCR products were obtained after nested PCR using the forward primer P276 (5′-CCGAGGAATTACTGGTCATCGCG-3′) and the reverse primer P204 (5′-GCTCTTCCATTGTTGGCTCGTC-3′). For those positive samples, genotyping RT-PCR started with the random hexamer or Iubs (5′-CCACACATACCAAGGCC-3′). The common reverse primers Iubs and nIubs (5′-CCAAGGCCATTTTACATA-3′) were used in both type I and type II genotyping PCR methods. For type I differentiation, the forward primers Iffs (5′-GTTTCAACCTAGAAAGCCTCAGAT-3′) and nIffles (5′-CCTAGAAAGCCTCAGATGAGTG-3′) were used in the first and nested PCR procedures, respectively, and subsequently generated 376-bp and 360-bp PCR products. For type II genotyping, the primers Icfs (5′-GCCTAGTATTATACCTGACTA-3′) and nIcfs (5′-CAGACCAAACTGGACTGTAC-3′) were added to the initial and nested PCR protocols, respectively, and 283-bp and 218-bp PCR products were obtained. PCR products were analyzed on 2% agarose gels.

### 2.4. Sequencing and Phylogenetic Analysis

The genotyping PCR products of type I and type II were sent to Tri-I biotech company (Taipei, Taiwan) for sequencing. For sequencing, BigDye^®^ Terminator v3.1 Cycle Sequencing Kit (Thermo Fisher Scientific, Waltham, MA, USA) was used and the data were analyzed by Applied Biosystems 3730 DNA Analyzer (Thermo Fisher Scientific). After the sequencing data became available, the sequencing chromatograms were carefully examined to ensure proper interpretation of the sequencing results. Sequences from both directions (forward and reverse) were checked to ensure consensus sequences. The sequences were submitted to the GenBank with accession numbers MW648553 to MW656208. The sequence alignment was created with the Clustal W method using CLC Main Workbench 20 (Qiagen, Germany). Phylogenetic trees were constructed with the neighbor-joining method using MEGA X software [18].

### 2.5. Statistical Analysis

The association of sex, breed, and age distribution in both the positive and negative groups was analyzed using Fisher’s exact test and the odds ratio with a 95% CI. The A/G ratio was analyzed by an unpaired Student’s t-test using Prism 9 (GraphPad, San Diego, CA, USA). *p* values smaller than 0.05 were considered significant.

### 2.6. Ethics Statement

The samples used in this study were derived during medical practice at the National Taiwan University Veterinary Hospital (NTUVH) and the use of the samples was approved by the cat owners and NTUVH with written consent. The ethics approval was waived according to the “Guideline for the Care and Use of Laboratory Animals” issued by the Council of Agriculture of Taiwan on 22 June 2018 and the official notice made by the Ministry of Health and Welfare of Taiwan on 5 July 2012 (document number: 1010265083).

## 3. Results

### 3.1. Detection of FCoV by IFA and RT-PCR

**Figure 1:**
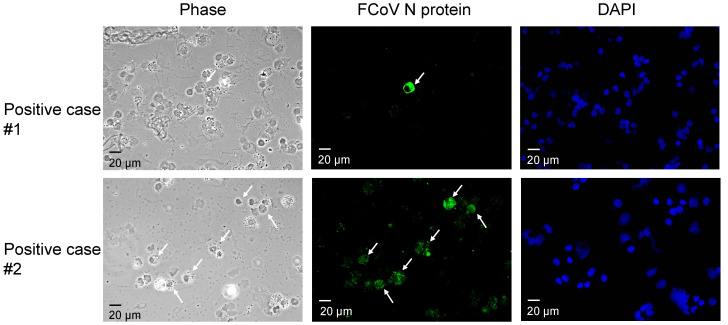
Fluorescence images of the specific cytoplasmic FCoV N protein signal in cats’ effusion samples. In the field of phase contrast, macrophages were identified. The arrows indicate the cytoplasmic antigen signals, and the nucleus was stained with DAPI.

Representative IFA images from two positive cases are shown in Figure 1, showing one or multiple macrophages with cytoplasmic FCoV N protein (green). False-positive images, such as those with FITC fluorescence located in the nucleus, were excluded from the analysis. Among the analyzed images, the IFA-negative image shows no cytoplasmic fluorescence signals in the macrophages. We considered cats with effusion that had positive IFA and RT-PCR results simultaneously to have a tentative diagnosis of FIP and classified them in the positive group. Among the cases analyzed in this study, there were no mismatches between the IFA and RT-PCR results. A total of 45 cats were categorized in the positive group, and 50 cats were classified as negative due to no detection of FCoV antigen by both the IFA and RT-PCR methods. The total positive rate was 47.4% (45/95) in this study.

### 3.2. Association of Positive Cases with Clinical Characteristics and A/G Ratio

**Figure 2:**
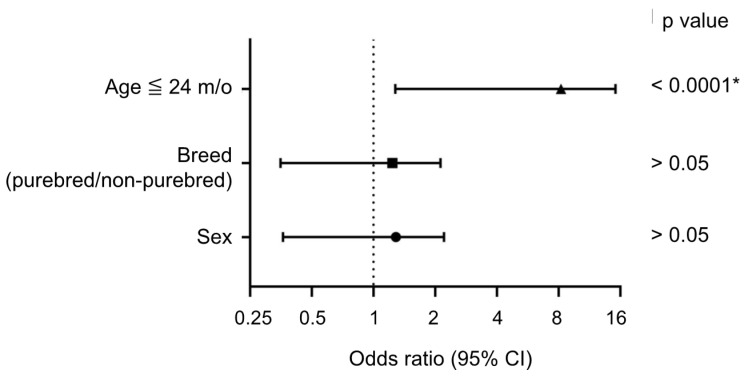
Odds ratio analysis for an included cat being diagnosed as FCoV positive based on age (≤24 m/o), breed, and sex. Odds ratios and 95% CIs were calculated using Fisher’s exact test. * *p* value < 0.05.

**Figure 3:**
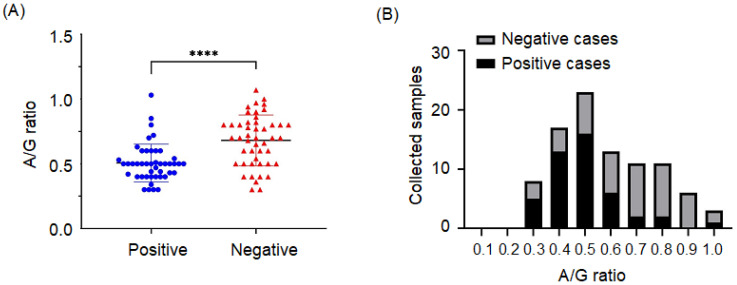
(**A**) A scatter dot plot demonstrating the A/G ratio of FCoV positive and negative cases. The *p* value was calculated based on an unpaired Student’s *t*-test. **** *p* < 0.0001. (**B**) FCoV positive and negative case distributions according to the A/G ratio.

The sex, breed, and age of the cats are summarized in Table 1; one cat in the positive group and one cat in the negative group had missing age data. In this study, positive rates in male and female cats were 48.08% (25/52) and 46.51% (20/43), respectively. The positive rate was 47.73% (21/44) in the purebred group and 47.06% (24/51) in the non-purebred group. The purebred cats in the positive group included four American shorthairs, four British shorthairs, two Scottish folds, two Russian blues, two Chinchillas, one Munchkin, and one Ragdoll. No significant association with either sex or breed is represented in Figure 2. Among the four age groups, the highest positive rate was 67.35% (33/49) in the 0~24-month-old group, and the highest negative rate was 15.63% (5/32) in the >73-month-old group. When the age cutoff value was further set as 24 m/o, the odds ratio of the age group ≤ 24 m/o was 6.19 (range from 2.54 to 16.00 in 95% CI) and showed a significant difference compared to the age group > 24 m/o (*p* < 0.0001). The A/G ratio in positive cases (*n* = 45) was significantly lower than the A/G ratio in negative cases (*n* = 47, as the A/G ratios of three cats were unavailable (Figure 3A,B). Most of the positive cases (88.9%) had an A/G ratio ranging from 0.3 to 0.5; however, five cases still had an A/G ratio ranging from 0.7 to 1.0.

### 3.3. FCoV Genotyping and Sequencing

A total of 32 out of 45 cases in the positive group had sufficient samples to perform FCoV genotyping, and all genotyping RT-PCR products were sequenced. These 32 positive samples were composed of 71.9% (23/32) type I strains only, and 28.1% (9/32) coinfection with type I and type II strains. Neither type II strains only nor untypable cases were noted in this study.

### 3.4. Phylogenetic Analysis

**Figure 4:**
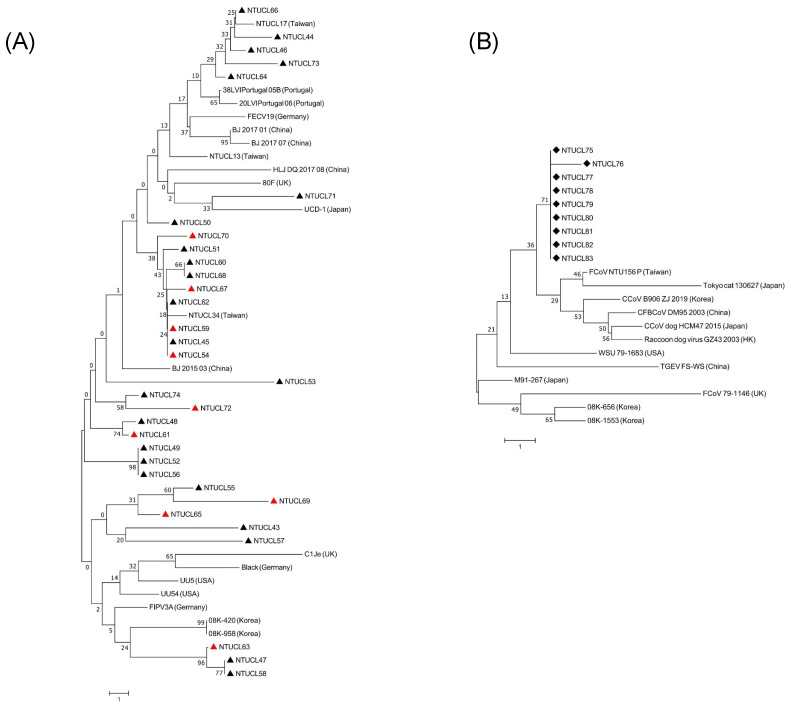
Phylogenetic analyses of FCoV isolates identified in effusion samples. (**A**) Phylogenetic tree of type I sequences. The triangle indicates the sequences generated in this study, the black triangle group (▲) had type I strains only, and the red triangle group (▲) had coinfected strains. (**B**) Phylogenetic tree of type II sequences. Diamonds (◆) indicate the type II sequences from the coinfection cases in this study. Neighbor-joining trees were constructed with 1000 bootstrap replicates.

A total of 32 type I sequences and 9 type II sequences were obtained in this study and uploaded to GenBank (see Table S1). Alignment of type I nucleotide sequences (110 nt) and protein sequences (36 amino acids) was created with our 32 sequences and 19 reference sequences. Although there was a high mutation rate in nucleotide changes, many of them were silent mutations and 56.3% (18/32) of sequences had no change in protein sequences. The other 13 sequences had 1–4 amino acid changes. In contrast, our type II sequences were relatively conserved, had a low mutation rate in nucleotides, and caused no change in proteins. Alignment of type II nucleotide sequences (202 nt) and peptide sequences (66 amino acids) was generated with our 9 sequences and 12 reference sequences. There was only one nucleotide change and no subsequent amino acid change in our sequences. The phylogenetic trees of type I and type II sequences are shown in Figure 4A,B, respectively. The type I internal sequence identity ranged from 85.5% to 100% and had 0 to 16 nucleotide changes. A similar sequence identity range (82.73–99.09%) was noted when compared to three Taiwan strains in 2018–2019 (NTUCL13, NTUCL17, NTUCL34). Similar wide sequence identity ranges were found when comparing our strains to other FCoV strains from different countries, which were 82.73–91.82% (one Japanese strain), 85.45–93.64% (two Korean strains), 87.27–94.55% (two American strains), 84.55–96.36% (three German strains), and 82.73–93.64% (two UK strains). Our type II sequences were close to Taiwan’s prototype strain (FCoV/NTU156/P/2007) identified in 2007. They also showed high similarity to the alphacoronaviruses from other species, including dogs, Chinese ferret badgers, raccoon dogs, and pigs. Sequence identity between FCoV and swine transmissible gastroenteritis virus, canine coronavirus, Chinese ferret badger coronavirus, and raccoon dog virus reached 97.52–99.01%, even higher than that of 79–1146 (94.55–95.05%), one of the oldest FCoV type II strains.

## 4. Discussion

The total positive rate of FCoV in effusion samples from cats in northern Taiwan was 47.4% in this study, which is within the range of the positive rate of 44–74.6% in different countries worldwide [19,20,21,22,23]. Some previous studies found that young cats, male cats, and several purebred cats were overrepresented in FIP cases [4]. In our study, young cats (≤2 y/o) were significantly overrepresented in the FCoV positive group, while breed and sex were not significantly different between the positive and negative groups. In addition, the A/G ratio was significantly lower in positive cases than in negative cases. Unity Jeffery reported that the A/G ratio is useful for ruling out FIP rather than ruling it in [24]. In our case, most cats in the positive group had an A/G ratio <0.6. However, there were still five cats in the positive group within the normal A/G ratio range (0.7~1.0). The exception cases remind us again that the A/G ratio is useful but cannot be the only evidence to rule out FIP.

There were 71.9% type I FCoV and 28.1% coinfection of type I and type II FCoV in this study. The genotyping data from different countries were collected for comparison; the type I strains only represented 97.5% in Malaysia [25], 95.8% in China [23], 83.3% in Japan [20], 86% in Australia [19], and 79% in Portugal [26]. Type II strains and coinfection cases represented small percentages—10.6% and 3.4% in Japan [20], 7% and 7% in Austria [27], 3.5% and 0% in Portugal [26], and 2.5% and 0% in Malaysia [25], respectively. Our FCoV genotype results even show a higher percentage of coinfection rate, and no type II FCoV-only cases were noted. These findings were surprising and we performed repeated assays to confirm our results. The reasons for the increase in coinfection cases and no type II strain single infection require further investigation. Around 13% of cats infected with type I FCoV become persistently infected cats [28], and therefore, one of the assumptions is that coinfected pet cats might have asymptomatic type I FCoV infection at first and then be infected with type II FCoV, which often leads to acute deterioration.

Type I FCoV strains in this study showed a high mutation rate in the partial S2 gene compared to that of type II FCoV. In addition, more than half of the nucleotide changes in the type I strains in this study remained silent, and the others led to 1–4 changes in proteins. In contrast, type II strains were closer to alphacoronaviruses from dogs, pigs, and other small mammals than other reference type II FCoV strains. Therefore, the CoV interactions of cats with pet dogs, small mammals, and even humans still require regular monitoring in the future. A recent study identified a novel canine–feline recombinant coronavirus that carries a type II FCoV-like S2 gene segment in samples from pneumonia patients of Malaysia, which raised concerns about the public health threat of animal CoVs [29].

There are several limitations of this study. First, we collected a small number of effusion samples. Since these samples came from different animal hospitals in the cities of northern Taiwan, they cannot represent the epidemiology of the total population in Taiwan, especially from the countryside, and this creates a selection bias. Second, we lacked the gold standard of histopathological diagnosis, but there was a study indicating that the IFA method has good specificity and sensitivity for diagnosis. Finally, our 110-bp type I sequences and 208-bp type II sequences were short and relatively conserved segments in the partial S2 gene; therefore, we could hardly know if the amino acid change led to any structural change. In addition, we had limited information about the survival time and the severity and progression of clinical presentations for further investigation of these strains.

## 5. Conclusions

Among 95 effusion samples from FIP-suspected cats, 47.5% were both IFA and PCR positive for FCoV. No significant association between positive rates and sex or breed was found, while the age group ≤ 24 m/o had significantly higher FCoV positive rates and had an odds ratio of 6.19 compared to the age group > 24 m/o. The A/G ratio in positive cases was significantly lower than the A/G ratio in negative cases. Among the positive cases, 71.9% (23/32) of cases had type I strains only, and 28.1% (9/32) of cases had coinfection. No single infections with type II strains were noted. The type I sequences had high diversity, whereas the type II sequences were conserved and even showed high similarity to the coronavirus from other species. This study demonstrates the latest analysis of FCoV infection cases in northern Taiwan.

## Figures and Tables

**Table 1:** Signalment summary of 95 cases in this study.

IFA and PCR	Sex		Breed		Age (m) ^#^			
	M	F	Purebred	Non-Purebred	0–24	25–48	49–72	>73
Positive (*n* = 45)	25	20	21	24	33	4	2	5
Negative (*n* = 50)	27	23	23	27	16	4	2	27
Total	52	43	44	51	49	8	4	32

^#^ Both groups have one missing data point of age.

## Data Availability

Data is contained within the article or supplementary material.

## Supplementary Materials

The following are available online at https://www.mdpi.com/article/10.3390/microorganisms9091801/s1, Table S1: FCoV partial spike sequences obtained in this study, Table S2: Reference sequences used in this study.
