# Supplementary material for: Feline Coronaviruses Identified in Feline Effusions in Suspected Cases of Feline Infectious Peritonitis
Source: Microorganisms. 2021 Aug 24;9(9):1801. doi: 10.3390/microorganisms9091801 (PMC8471460; doi:10.3390/microorganisms9091801)
Supplement: Supplementary file 1 [file microorganisms-09-01801-s001.zip › microorganisms-1260364-supplementary.pdf]

**Table S1.** FCoV partial spike sequences obtained in this study.

| Virus Name | GenBank Accession Number | Sample Type | Collection Date | FCoV Genotype |
|---|---|---|---|---|
| NTUCL43 | MW648553 | ascites | Jan-2019 | I |
| NTUCL44 | MW648554 | ascites | Feb-2019 | I |
| NTUCL45 | MW648555 | ascites | Apr-2019 | I |
| NTUCL46 | MW648556 | ascites | June-2019 | I |
| NTUCL47 | MW648557 | ascites | July-2019 | I |
| NTUCL48 | MW648558 | ascites | July-2019 | I |
| NTUCL49 | MW648559 | pleural effusion | July-2019 | I |
| NTUCL50 | MW648560 | ascites | July-2019 | I |
| NTUCL51 | MW648561 | pleural effusion | Aug-2019 | I |
| NTUCL52 | MW648562 | ascites | Aug-2019 | I |
| NTUCL53 | MW648563 | ascites | Oct-2019 | I |
| NTUCL54 | MW648564 | pleural effusion | Nov-2019 | I |
| NTUCL55 | MW648565 | ascites | Dec-2019 | I |
| NTUCL56 | MW648566 | pleural effusion | Dec-2019 | I |
| NTUCL57 | MW648567 | ascites | Jan-2020 | I |
| NTUCL58 | MW648568 | ascites | Jan-2020 | I |
| NTUCL59 | MW648569 | ascites | Apr-2020 | I |
| NTUCL60 | MW648570 | ascites | Apr-2020 | I |
| NTUCL61 | MW648571 | pleural effusion | Apr-2020 | I |
| NTUCL62 | MW648572 | pleural effusion | May-2020 | I |
| NTUCL63 | MW648573 | ascites | May-2020 | I |
| NTUCL64 | MW648574 | pleural effusion | June-2020 | I |
| NTUCL65 | MW648575 | pleural effusion | June-2020 | I |
| NTUCL66 | MW648576 | ascites | June-2020 | I |
| NTUCL67 | MW648577 | pleural effusion | July-2020 | I |
| NTUCL68 | MW648578 | pleural effusion | July-2020 | I |
| NTUCL69 | MW648579 | ascites | July-2020 | I |
| NTUCL70 | MW648580 | ascites | Aug-2020 | I |
| NTUCL71 | MW648581 | ascites | Sep-2020 | I |
| NTUCL72 | MW648582 | ascites | Sep-2020 | I |
| NTUCL73 | MW648583 | ascites | Oct-2020 | I |
| NTUCL74 | MW648584 | ascites | Oct-2020 | I |
| NTUCL75 | MW656200 | pleural effusion | Nov-2019 | II |
| NTUCL76 | MW656201 | ascites | Apr-2020 | II |
| NTUCL77 | MW656202 | pleural effusion | Apr-2020 | II |
| NTUCL78 | MW656203 | ascites | May-2020 | II |
| NTUCL79 | MW656204 | pleural effusion | June-2020 | II |
| NTUCL80 | MW656205 | pleural effusion | July-2020 | II |
| NTUCL81 | MW656206 | ascites | July-2020 | II |
| NTUCL82 | MW656207 | ascites | Aug-2020 | II |
| NTUCL83 | MW656208 | ascites | Sep-2020 | II |

**Table S2.** Reference sequences used in this study.

| Strain | Host Species | FCoV Genotype | Location | Genbank Accession Number |
|---|---|---|---|---|
| NTUCL34 | *Felis catus* | I | Taiwan | MK736801 |
| NTUCL13 | *Felis catus* | I | Taiwan | MK736789 |
| NTUCL17 | *Felis catus* | I | Taiwan | MK736792 |
| BJ/2017/01 | *Felis catus* | I | China | MG892399 |
| BJ/2017/07 | *Felis catus* | I | China | MG892400 |
| BJ/2015/03 | *Felis catus* | I | China | MG016685 |
| HLJ/DQ/2017/08 | *Felis catus* | I | China | MG892408 |
| UCD-1 | *Felis catus* | I | Japan | AB088222 |
| 08K-958 | *Felis catus* | I | Korea | JN654404 |
| 08K-420 | *Felis catus* | I | Korea | JN654401 |
| UU5 | *Felis catus* | I | USA | FJ938056 |
| UU54 | *Felis catus* | I | USA | JN183883 |
| 20LVIPortugal/06 | *Felis catus* | I | Portugal | EU327712 |
| 38LVIPortugal/05B | *Felis catus* | I | Portugal | EU327731 |
| Black | *Felis catus* | I | Germany | EU186072 |
| FECV19 | *Felis catus* | I | Germany | KJ665866 |
| FIPV3A | *Felis catus* | I | Germany | KJ665871 |
| 80F | *Felis catus* | I | UK | KP143511 |
| C1Je | *Felis catus* | I | UK | DQ848678 |
| FCoV/NTU156/P/2007 | *Felis catus* | II | Taiwan | EU513388 |
| M91-267 | *Felis catus* | II | Japan | AB781788 |
| Tokyo/cat/130627 | *Felis catus* | II | Japan | AB907634 |
| 08K-1553 | *Felis catus* | II | Korea | JN654416 |
| 08K-656 | *Felis catus* | II | Korea | JN654413 |
| WSU 79-1683 | *Felis catus* | II | USA | JN634064 |
| 79-1146 | *Felis catus* | II | UK | DQ010921 |
| CFBCoV/DM95/2003 | *Malva moschata* | | China | EF192156 |
| CCoV B906_ZJ_2019 | *Canis lupus* | | China | MT114554 |
| TGEV FS-WS | *Sus scrofa* | | China | MK272773 |
| GZ43/2003 | *Nyctereutes procyonoides* | | Hong Kong | EF192155 |
| CCoV/dog/HCM47/2015 | *Canis lupus* | | Japan | LC190907 |
